# Supplementary material for: Early postoperative complications following laparoscopic-assisted modified Soave procedure for Hirschsprung’s disease: Incidence, Clavien-Dindo classification, and risk factor analysis
Source: PLoS One. 2025 Sep 22;20(9):e0332457. doi: 10.1371/journal.pone.0332457 (PMC12453232; doi:10.1371/journal.pone.0332457)
Supplement: Attachment — Submitted filename: Response to Reviewers.docx [file pone.0332457.s003.docx]

**Dear Reviewer,**

We are very grateful to the reviewers for their constructive comments and suggestions. We have revised the manuscript accordingly, as indicated in red font in all revised manuscripts. In addition, our point-by-point response to these comments is listed below this letter.

We hope that our revised manuscript will be successfully published and look forward to hearing from you soon.

With best wishes,

Yours sincerely,

Xianhui Shang

**Response to Reviewer 1**
First of all, we would like to express our sincere gratitude to the reviewers for their constructive and positive comments.
1. The classification of HSCR should follow standard terminology like short segment disease, long segment disease etc; rather than common type.
**Response: Thank you very much for your valuable comments.**

In response to the comment on HSCR classification terminology:

We have replaced all instances of “common type” and “long-segment type” with the internationally accepted terms “short-segment disease” and “long-segment disease.”
2. This study focused on surgical related complications and complications like laryngeal edema should be excluded. Indeed, during the risk factors analysis, I think it is more useful if the analysis focused on grade III or above complications
**Response: We sincerely appreciate your helpful suggestions.**

Regarding the inclusion of non-surgical complications such as laryngeal edema:

We have excluded the case of laryngeal edema from all statistical analysis and complication counts. Additionally, we have added a focused analysis on Clavien-Dindo grade III and above complications to highlight surgically relevant adverse events(Page9, Table 3).
3. What are the reasons for creating stoma before operation?
**Response: Thank you for your thoughtful feedback.**

About the indications for preoperative stoma:

In the “Materials and Methods” section, we have expanded the explanation of indications for preoperative stoma creation, including severe recurrent HAEC, poor preoperative nutritional status (e.g., hypoalbuminemia, anemia), and suspicion of long-segment or total colonic aganglionosis based on imaging(Page3-4, Lines 88-95).
4.There is no need to insert a table explaining CD classification. This would save some space and most readers will know about this classification system
**Response: We are grateful for your kind review.**

Concerning the inclusion of a table for Clavien-Dindo classification:

As suggested, we have removed the Clavien-Dindo classification table to streamline the manuscript. The classification criteria are now briefly described in the text of the Methods section(Page 6, Lines 142-155).
5. What were the reasons for low albumin and haemoglobin prior to the pullthrough procedure?
**Response: Thank you for taking the time to evaluate our work.**

On the causes of hypoalbuminemia and anemia:

In the Discussion section, we have elaborated on the causes , explaining that most patients presented with chronic constipation, leading to nutritional deficiencies and impaired absorption due to HAEC , which contributed to the observed preoperative hypoalbuminemia and anemia(Page 16-17, Lines 347-349, 355-357, 367-371).

## 6. How many patients required further operation to manage the complications? **Response: We appreciate your insightful input.**

## About reoperations following complications:

We have added a new sentence to the Results section , stating that six patients with Clavien-Dindo grade IIIb or higher complications underwent additional surgeries , including adhesiolysis, stoma creation, and rectal retraction correction . This information is also reflected in the discussion(Page 8-9, Lines 204-210, Table 3).

7. The title 'Laparoscopic-Assisted Modified Soave Procedure for the Treatment of Early Postoperative Complications in Hirschsprung's Disease: Clavien-Dindo Classification and Influencing Factors' is mis-leading. This title gives the impression that lap assisted modified procedure is used for treating the complications in HSCR. Please revise it.

**Response: Many thanks for your constructive advice.**

Revised title as recommended:

We have changed the manuscript title to: “Early Postoperative Complications Following Laparoscopic-Assisted Modified Soave Procedure for Hirschsprung’s Disease: Incidence, Clavien-Dindo Classification, and Risk Factor Analysis” to avoid any implication that the procedure was used to treat complications.

**Response to Reviewer 2:**

We sincerely thank the reviewer for the thoughtful comments and constructive suggestions. Below we provide our point-by-point responses:

1.The findings are not new, lack novelty and mostly confirmatory. The authors should clarify the novel aspect of this study compared to existing literatures and discuss how these findings could influence clinical practice or patient management strategies.

**Response:** Thank you for your valuable feedback.

While we acknowledge that laparoscopic-assisted modified Soave procedures are well-established, our study offers the following **novel contributions**:

**Application of the Clavien-Dindo (CD) classification system specifically for early postoperative complications in pediatric HSCR patients**—this has rarely been systematically applied in previous HSCR studies.

**Identification of hypoalbuminemia and preoperative stoma as independent risk factors** for early complications in this population, based on multivariate analysis, which may provide actionable targets for perioperative optimization.

Our findings **emphasize the need for individualized nutritional intervention and improved perioperative assessment** in patients undergoing definitive surgery for HSCR, which may influence protocols for surgical timing, preoperative workup, and resource allocation in clinical practice.

We have revised the **Discussion** and **Conclusion** sections to better highlight these clinical implications and clarify the contribution of our study relative to existing literature(Page 12,17 Lines 251-255, 382-389).

2. The authors may also consider to show the data of short segment type and long segment type separately and discuss.

**Response:** Thank you for this suggestion.

We agree that stratified analysis may provide additional insights. We have now included **subgroup data comparing short-segment vs. long-segment HSCR** in Table 2 and the Results section, and briefly discussed their respective complication profiles in the Discussion. While the complication rates between these groups were not statistically significant, our data suggest a trend toward more complex presentations in long-segment cases, which warrants further investigation in larger cohorts(Page 13, Lines 262-268).

3. If the authors provides a table summarizing the previous published references describing early postoperative complications after lap Soave, it would be educative.

**Response:** We appreciate this excellent suggestion.

Accordingly, we have added a new table (**Table 2**) summarizing key published studies reporting early postoperative complications following laparoscopic Soave procedures. This comparative summary highlights the complication rates, follow-up durations, and reported risk factors across studies, and we have referred to it in the Discussion section to place our findings in context.
