# Supplementary material for: Early postoperative complications following laparoscopic-assisted modified Soave procedure for Hirschsprung’s disease: Incidence, Clavien-Dindo classification, and risk factor analysis
Source: PLoS One. 2025 Sep 22;20(9):e0332457. doi: 10.1371/journal.pone.0332457 (PMC12453232; doi:10.1371/journal.pone.0332457)
Supplement: Attachment — Submitted filename: Response_to_Reviewers_auresp_2.docx [file pone.0332457.s004.docx]

**Dear Reviewers,**

We sincerely thank you for the valuable time and effort you have dedicated to reviewing our manuscript. We greatly appreciate the constructive feedback and thoughtful suggestions provided during the review process. These insights have helped us significantly improve the clarity, rigor, and overall quality of our work.

In this revised version, we have carefully addressed all the comments raised by both reviewers. Key modifications include:

**·Clarification of the study’s novelty and contribution**: We emphasized the first systematic application of the Clavien-Dindo classification to early postoperative complications in pediatric HSCR patients undergoing laparoscopic-assisted modified Soave procedures, along with the identification of hypoalbuminemia as an independent risk factor.

**·Revision of the Results section**: We restructured and streamlined the language to eliminate redundancy and improve readability, as advised by Reviewer 2.

**·Correction of Table formatting**: All Chinese-language entries have been converted to standard English, and explanatory notes have been added to improve clarity.

**·Expansion of literature scope**: In response to concerns about reference diversity, we have supplemented Table 2 with representative international studies, including those by Beltman et al., Ahmad et al., and Gershon et al., and we have discussed their relevance explicitly in the updated Discussion section.

All revisions are clearly highlighted in red font in the tracked manuscript version.

A detailed, point-by-point response to each reviewer comment is included below this letter for your reference.

We sincerely hope that the revised manuscript now meets the standards for publication and contributes meaningfully to the field. We are grateful for your consideration and look forward to your feedback.

With our warmest regards,
**Yours sincerely,**
**Xianhui Shang**
(On behalf of all co-authors)

### ****Question 1: If you feel the authors have adequately addressed your comments raised in a previous round of review and you feel that this manuscript is now acceptable for publication, you may indicate that here and submit an “Accept” recommendation.****

**Reviewer 1:** All comments have been addressed.

**Response:**
We sincerely thank Reviewer 1 for the positive evaluation. We are pleased to learn that all prior comments have been considered adequately addressed. We truly appreciate your support and guidance.

**Reviewer 2:** No response.

**Response:**
Although Reviewer 2 did not provide an explicit response to this item, we have carefully revised the manuscript to address each of their prior concerns — including issues regarding novelty, language redundancy, table formatting, and literature source diversity — with detailed explanations and substantive edits. We respectfully request the editorial team to take these improvements into account in making a final decision.

**Question 2: Is the manuscript technically sound, and do the data support the conclusions?**

**Response:**
This study is based on a complete retrospective dataset of 112 patients, with all data derived from authentic inpatient medical records and reviewed under ethical approval (Approval No.: KLLY-2020-039). All variables were clearly defined, and both univariate and multivariate logistic regression analyses were conducted to ensure methodological rigor. We believe the data adequately support the study’s conclusions, and the limitations have been transparently addressed in the relevant section.

### ****Question 3: Has the statistical analysis been performed appropriately and rigorously?****

**Response:**
Statistical analysis was conducted using SPSS 18.0. Data distribution was presented with appropriate descriptive statistics (mean ± standard deviation, median and interquartile range). Significance testing was performed using two-tailed p-values, and the multivariate logistic regression clearly presents odds ratios (OR) with 95% confidence intervals (CI). We have revised the “Statistical Methods” and “Results” sections to further enhance the clarity and rigor of the analysis as per reviewer suggestions.

### ****Question 4: Have the authors made all data underlying the findings in their manuscript fully available?****

**Response:**
We have prepared and uploaded the core de-identified dataset as a **Supporting Information** Excel file (see Appendix Data S1). This file includes all analytical variables, types of postoperative complications, and Clavien-Dindo classification entries, in full compliance with the PLOS Data Availability Policy.

### ****Question 5: Is the manuscript presented in an intelligible fashion and written in standard English?****

**Response:**
Thank you for this important reminder. In this revision, the manuscript underwent professional language editing by a native English-speaking medical editor. We specifically refined the “Results” and “Discussion” sections to eliminate redundancy, unify terminology, and streamline sentence structures, ensuring that the manuscript now meets the clarity and language standards expected for international publication.

### ****Question 6: Reviewer Comments****

**Reviewer 1:** No additional comments.

**Response:**
We appreciate the thoughtful suggestions from Reviewer 1 provided in the previous round. Although no further comments were submitted this round, we have carefully verified and implemented all prior recommendations, ensuring that every revision has been addressed in detail to uphold scientific rigor and manuscript quality.

**Reviewer 2:**
This manuscript presents a single-institution analysis of surgical outcomes and complications in patients with Hirschsprung’s disease. Although the authors applied the Clavien-Dindo classification to grade postoperative complications, the study lacks novelty, as previously pointed out by Reviewer 2. In addition, the **Results** section contains repetitive and redundant sentence structures. Some table entries (e.g., Table 2) are written in Chinese, which affects the overall clarity of the manuscript. Furthermore, all references cited in Table 2 are from domestic Chinese sources, and the authors did not explain the rationale for selecting these particular studies. Addressing these issues would significantly improve the quality and impact of the manuscript.

**Comment 1:**
**“This manuscript presents a single-institution analysis of surgical outcomes and complications in patients with Hirschsprung’s disease. Although the authors applied the Clavien-Dindo classification to grade postoperative complications, the study lacks novelty, as previously pointed out by Reviewer 2.”**

**Response:**
We sincerely thank you for raising the important issue of the study's novelty. While this is indeed a single-center retrospective analysis, we believe the following aspects distinguish our work and offer valuable contributions to the field:

1. **First systematic application of the Clavien-Dindo classification to postoperative complications in children with HSCR**
   Although the Clavien-Dindo system is widely used in adult surgical complication assessment, its systematic application in pediatric Hirschsprung’s disease (HSCR)—particularly following laparoscopic-assisted modified Soave procedures—has not been thoroughly reported. Our study employs rigorous data analysis to objectively classify early complications by severity using the CD system, which remains rare in both domestic and international literature.
2. **First identification of preoperative hypoalbuminemia as an independent high-risk factor**
   While previous studies have generally associated nutritional status with surgical outcomes, our study—through multivariate logistic regression—demonstrates for the first time that hypoalbuminemia (<35 g/L) is an independent risk factor for early postoperative complications (OR = 9.14, P < 0.001), offering a direct evidence base for targeted preoperative nutritional intervention.
3. **Relatively large sample size and long study duration**
   Our analysis includes 112 pediatric patients treated over a 10-year span (2010–2020), with detailed perioperative data, surgical variables, complication classifications, and CD grading. Compared to prior studies that often focus on isolated complications or smaller datasets, our work provides more comprehensive and structured insights.
4. **Clear distinction and complementary value compared to existing domestic studies**
   In response to concerns about the predominance of domestic references, we have revised Table 2 to include representative international studies (e.g., Ahmad et al., Beltman et al.), and clarified in the Discussion how our methodology, classification system, and population characteristics complement the global literature.
5. **Clinical significance and practical value**
   Beyond reporting complication incidence, our study emphasizes actionable clinical strategies—such as the correction of anemia and hypoalbuminemia—to reduce complication rates. These findings carry practical value for improving perioperative care in HSCR patients.

Should the editorial team deem it necessary, we would be glad to further highlight these novel aspects in the revised Discussion section, particularly the implications for preoperative nutritional optimization and the recommendation of albumin screening as part of routine risk assessment.

We have already emphasized these contributions in the revised Introduction and Discussion sections to ensure they are clearly recognized by readers and reviewers(Page 3-4, 14-15, 20 Lines 71-87, 95-99, 270-287, 292-294, 299-303, 434-441).

**Comment 2:**
**“The Results section contains repetitive and redundant sentence structures.”**

**Response:**
We have thoroughly revised the Results section to remove redundancy and improve narrative clarity. Specifically:

·Redundant structures (e.g., “...18 patients developed complications, with 21 events... three patients had two complications each.”) have been consolidated and logically rephrased;

·Terminology has been standardized (e.g., consistently using “complication group” vs. “non-complication group”);

·Sentence structures have been simplified to avoid repetition such as “Patients with... were found to...”.

These changes have resulted in a more concise and readable results presentation(Page 7-12 Lines 186-206, 217-231, 242-251).

**Comment 3:**
**“Some table entries (e.g., Table 2) are written in Chinese, which affects the overall clarity of the manuscript.”**

**Response:**
We have fully revised Table 2 to ensure all terminology and entries are presented in standard English. Key revisions include:

Converting all time units (e.g., “6个月”, “24个月”) into English (“6 months”, “24 months”);

Adding a note for abbreviations (e.g., “CD = Clavien-Dindo”) to enhance reader comprehension.

The updated table now adheres to international publication standards and improves clarity for global readers(Page 9 Table 2).

**Comment 4:**
**“All references cited in Table 2 are from domestic Chinese sources, and the authors did not explain the rationale for selecting these studies.”**

**Response:**
We sincerely acknowledge this important point and have revised the manuscript accordingly. Our original selection of domestic studies was based on the following justifications:

**Comparability**: The selected studies reflect similar surgical approaches, resource settings, and patient profiles to our center, allowing for more meaningful benchmarking of complication rates;

**Lack of global data using the CD system in HSCR**: There is a notable absence of international studies systematically applying the Clavien-Dindo classification to early postoperative complications in HSCR. Most global literature focuses on long-term outcomes or functional assessment.

In response to your comment, we have now included internationally representative studies in the revised Table 2 and discussion, such as:

·Beltman et al., J Pediatr Surg, 2021 – CD classification use in transanal pull-through;

**·**Ahmad et al., Semin Pediatr Surg, 2022 – Management of suboptimal outcomes in HSCR;

·Gershon et al., World J Clin Pediatr, 2023 – Comprehensive review on HAEC.

We have also added a paragraph in the Discussion explicitly explaining the rationale for literature selection and the value of combining domestic and international comparisons to strengthen the paper’s generalizability(Page 15 Lines 306-320).

**Comment 5:**
**“Addressing these issues would significantly improve the quality and impact of the manuscript.”**

**Response:**
We fully agree with your conclusion. Through meticulous revision of the issues you raised—including language clarity, table formatting, literature diversity, and articulation of our study’s significance—we believe the manuscript is now greatly improved in clarity, rigor, and relevance to an international audience. We are deeply grateful for your constructive feedback, which has been instrumental in enhancing the manuscript’s quality and impact.
