# Supplementary material for: Early postoperative complications following laparoscopic-assisted modified Soave procedure for Hirschsprung’s disease: Incidence, Clavien-Dindo classification, and risk factor analysis
Source: PLoS One. 2025 Sep 22;20(9):e0332457. doi: 10.1371/journal.pone.0332457 (PMC12453232; doi:10.1371/journal.pone.0332457)
Supplement: Attachment — Submitted filename: Response_to_Reviewers_auresp_3.docx [file pone.0332457.s005.docx]

**Dear Reviewer 3,**

We sincerely appreciate your careful review and the constructive comments you provided. Your feedback has been invaluable in improving the quality and clarity of our manuscript.

We have carefully revised the manuscript in response to your suggestions. All changes are clearly marked in **red font** in the revised version. Additionally, a detailed **point-by-point response** addressing each of your comments is provided below this letter.

We are truly grateful for your positive and thoughtful remarks, which have greatly contributed to the refinement of our study. We hope that our revised manuscript meets the standards of the journal and will be favorably considered for publication.

Thank you once again for your time and effort in reviewing our work.

With best regards,
**Xianhui Shang**
On behalf of all co-authors

**Replies to Reviewer 3**

**Major comments**

1. the Results section still contains repetitive sentences，The Result section has titles 1,3,4, but no 2. It is suggested the figure in 3 be integrated into 1, and the data in the table should no longer be described in words.

**Response:**

**We sincerely thank the reviewer for the careful observation. In the revised version, we have reorganized the Results section to ensure logical consistency and avoid redundancy. Specifically, we have added the missing subheading “2” to maintain proper sequence, integrated the figure originally placed in section 3 into section 1 as suggested, and removed repetitive textual descriptions of the tabulated data. These changes have improved the clarity and conciseness of the results presentation**(Page 7-12 Lines 188-227).

2. There are too many descriptions of the seven causes of early postoperative complications in the discussion. Some in textbooks or previous literature are suggested to be deleted, and only the relevant results of this study should be discussed.

**Response:**

**We sincerely thank the reviewer for the insightful comment. We have carefully reviewed the Discussion section and reduced the descriptions related to the seven causes of early postoperative complications. Specifically, we deleted portions that overlapped with textbook knowledge or previously published literature, retaining only the content directly relevant to the findings of our study, and made corresponding adjustments to the references. We believe that these revisions make the discussion more focused on our study results and enhance the specificity and readability of the manuscript**(Page 12-14 Lines 228-288).

3. The Limitations section "a single center with a small sample size (428.429 lines)" contradicts "its relatively large sample size (291 lines)" in the discussion.

**Response:**

**We thank the reviewer for carefully pointing out the inconsistency between the statements on sample size in the Discussion and Limitations sections. In the revised manuscript, we have unified and clarified the wording: the phrase “relatively large sample size” has been removed, and we now provide an objective description of the actual scale and design of the study. Specifically, we report only the factual information that 112 patients were included between 2010 and 2020, without making subjective comparisons with previous literature. In the Limitations section, we consistently state that this is a single-center retrospective study with a limited sample size, and we emphasize the potential risks of limited applicability and information bias inherent to a single-center design and a 30-day complication assessment window. We believe these revisions eliminate the inconsistency and more accurately reflect the scope and limitations of the study**(Page 14 Lines 277-280).

4. The conclusion in the Abstract section suggests deleting "Perioperative correction of anemia" because the Multivariate analysis was not significant.

**Response:**

**We sincerely appreciate the reviewer’s thoughtful suggestion. We have carefully reviewed the conclusion of the abstract and revised it according to the results of the multivariate analysis. Specifically, the statement “perioperative correction of anemia” has been removed, and only conclusions supported by statistically significant findings have been retained. This ensures that the abstract accurately reflects the results of the multivariate analysis**(Page 2 Lines 56-58).

5. Important descriptions in the Introduction section need to be marked with references. For example, "its structured use in pediatric HSCR patients remains extremely limited in both domestic and international (lines 75 and 76)" .

**Response:**

**We sincerely thank the reviewer for the valuable comment. In the Introduction, we have added a reference to support the statement that “its structured application in pediatric HSCR patients remains extremely limited both domestically and internationally,” thereby enhancing the reliability and completeness of the discussion**(Page 3 Lines 76-78; Page 15 Lines 300-303).
